# Controversial effects of metformin on human physiology and pathophysiology

**DOI:** 10.3389/fphar.2026.1799374

**Published:** 2026-04-24

**Authors:** Shuo Fang, Qirui Liu, Yi Lu, Jian Zhang

**Affiliations:** 1 Department of Clinical Medicine, School of Medicine, Southern University of Science and Technology, Shenzhen, China; 2 Department of Human Cell Biology and Genetics, School of Medicine, Southern University of Science and Technology, Shenzhen, China; 3 Joint Laboratory of Guangdong-Hong Kong Universities for Vascular Homeostasis and Diseases, SUSTech Homeostatic Medicine Institute, School of Medicine, Southern University of Science and Technology, Shenzhen, China; 4 Clinical Research Center, The First People’s Hospital of Foshan (The Affiliated Foshan Hospital of Southern University of Science and Technology), School of Medicine, Southern University of Science and Technology, Shenzhen, Guangdong, China

**Keywords:** AMPK, controversial effect, metformin, mTOR, pathophysiology

## Abstract

Metformin is the first-line oral antihyperglycemic agent for type 2 diabetes mellitus (T2DM), with over 150 million individuals worldwide receiving treatment annually. Although synthesized in 1922, its clinical adoption began in the 1950s, and decades of widespread use have firmly established its efficacy in glycemic control and metabolic improvement. Nevertheless, growing evidence indicates that metformin exerts pleiotropic effects beyond glucose homeostasis—modulating neurocognitive function, gastrointestinal physiology, reproductive endocrinology, and cellular aging pathways. Many of these effects remain incompletely characterized, mechanistically ambiguous, or clinically inconsistent across populations. This review systematically synthesizes recent (2014–2024) preclinical and clinical evidence from PubMed and Google Scholar to critically evaluate the contested physiological and pathophysiological actions of metformin. We distinguish robustly replicated findings from preliminary or contradictory observations, clarify dose- and context-dependent mechanisms (e.g., mitochondrial vs. lysosomal AMPK activation), and highlight knowledge gaps impeding safe, personalized application. Emphasis is placed on reconciling mechanistic insights with real-world therapeutic outcomes—particularly regarding long-term safety, interindividual variability in pharmacokinetics (e.g., OCT/MATE transporter polymorphisms), and the need for prospective studies integrating multi-omics profiling and extended follow-up.

## Graphical Abstract

**Figure:**
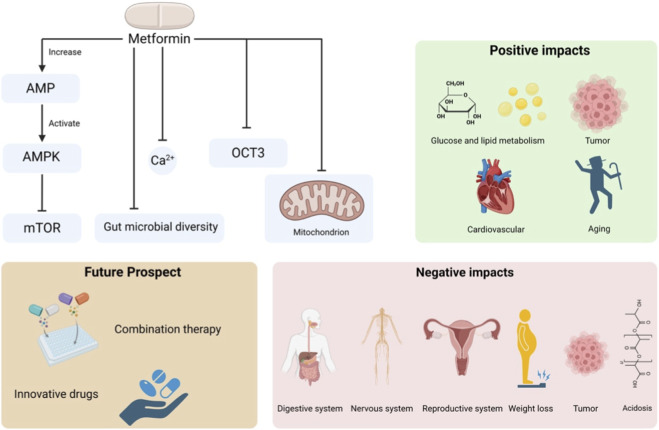

## 1 Introduction

Metformin is currently the first-line drug for treating type 2 diabetes mellitus (T2DM). In addition to its hypoglycemic effect, the effects on nutrient metabolism, cardiovascular disease, and cancer have garnered research interest (Foretz et al., 2014). Furthermore, metformin exerts a certain effect in treating Alzheimer’s disease (AD). Its therapeutic effects on various pathophysiological mechanisms of AD, including the generation and clearance of amyloid-beta (Aβ), tau phosphorylation, and neuroinflammation, have not been fully established (Khezri et al., 2022). Moreover, the side effects of metformin on the human body have not been sufficiently investigated. Given this research gap, the objectives of this article were to summarize the available research on the positive effects of treatment with metformin and discuss any controversy regarding its effects at the physiological and pathophysiological levels. This compiled information may be useful to patients treated with the drug for extended periods of time.

Multiple drug transporters are involved in the absorption of metformin in the human body, with organic cation transporters (OCTs) playing a vital role (Ciarimboli, 2008). Particularly, OCT3 is chiefly responsible for the absorption of the drug in the intestines (Muller et al., 2005; Yang et al., 2021). Following entry into the bloodstream, the drug must reach the liver to exert its therapeutic effects; this process is predominantly mediated by OCT1 located on the hepatocyte basement membrane. In the kidneys, metformin is transported from the bloodstream into renal tubular epithelial cells via OCT2 and is ultimately excreted in urine via the multidrug and toxic compound efflux transporters (MATE1/2-K) (Becker et al., 2009). Under standard dosing regimens (500–2,550 mg/day), the steady-state plasma concentration of metformin is typically <1 μg/mL. Peak plasma concentration is attained approximately 2.5 h after administration, and its elimination half-life is approximately 5 h.

## 2 Important molecular mechanisms related to metformin

**FIGURE 1:**
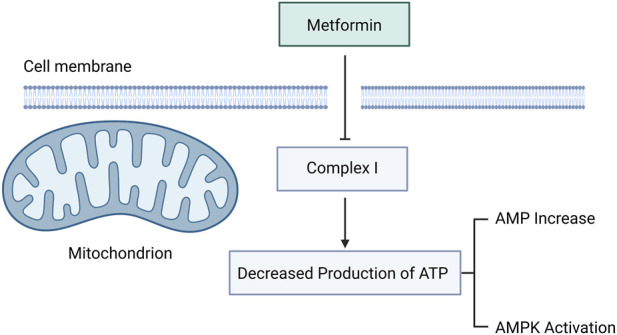
Molecular mechanism of metformin. Metformin reduces ATP synthesis by inhibiting mitochondrial complex I, leading to increased AMP levels and activation of AMP-activated protein kinase (AMPK).

Metformin’s pharmacological effects converge largely on AMP-activated protein kinase (AMPK) activation—but through multiple, concentration-dependent, and compartmentally distinct pathways. At clinically relevant concentrations (≤5 μM), metformin accumulates in lysosomes via the proton gradient and binds presenilin enhancer 2 (PEN2), thereby inhibiting vacuolar H^+^-ATPase (v-ATPase) and triggering AMPK activation independently of mitochondrial perturbation (Ma et al., 2022). At intermediate concentrations (∼2 mM, often used *in vitro* but exceeding typical plasma levels), metformin engages the AXIN-LKB1 scaffold complex to activate AMPK in an LKB1-dependent manner (Woods et al., 2003). Only at supraphysiological concentrations (>5 mM)—unattainable in human plasma under standard dosing—does metformin robustly inhibit mitochondrial respiratory chain complex I, reducing ATP synthesis and elevating AMP:ATP ratios (Figure 1) (El-Mir et al., 2000; Owen et al., 2000; Fontaine, 2018). Supporting this hierarchy, Wang et al. (2019) demonstrated that metformin downregulates adenylosuccinate synthetase (AdSS) and adenylosuccinate lyase (AdSL) mRNA expression by ∼60%, impairing *de novo* purine synthesis and contributing to AMP accumulation. Further, Dutta et al. (2023) identified AMP deaminase (AMPD) inhibition as a complementary mechanism elevating AMP, corroborated by Ouyang et al. (2011), who showed abolished AMP accumulation and glucose uptake in AMPD-knockout models. Collectively, these findings refute a singular “mitochondrial-only” paradigm and instead support a tiered, context-sensitive model of AMPK activation—where lysosomal, cytosolic, and mitochondrial mechanisms operate sequentially as drug concentration increases.

## 3 Physiological benefits of metformin: evidence and nuance

Metformin exerts a broad spectrum of beneficial effects beyond glycemic control, influencing glucose and lipid metabolism, cardiovascular function, and even the aging process. These effects are mediated through a combination of direct molecular mechanisms and indirect actions involving the gut microbiota, systemic metabolism, and cellular signaling pathways.

### 3.1 Regulation of glucose metabolism

Metformin suppresses hepatic gluconeogenesis primarily by reducing cellular energy charge: inhibition of mitochondrial complex I (at high concentrations) or lysosomal v-ATPase (at therapeutic concentrations) lowers ATP availability, thereby constraining this ATP-intensive process. Concurrently, elevated AMP directly inhibits fructose-1,6-bisphosphatase and adenylate cyclase—key regulators of gluconeogenic flux (Foretz et al., 2023). Importantly, gut microbiota mediate a substantial portion of metformin’s glucose-lowering effect. Clinical and murine studies consistently report increased abundance of Akkermansia muciniphila following metformin treatment (Ruan et al., 2023). This bacterium enhances glucose tolerance, ameliorates insulin resistance, and reinforces intestinal mucosal barrier function—effects recapitulated in germ-free mice colonized with A. muciniphila (Bednarska and Haberg, 2025). Thus, metformin’s antidiabetic action reflects a tripartite interaction among host transporters, cellular energy sensors, and microbial ecology.

### 3.2 Modulation of lipid metabolism

In addition to its suppressive effect on gluconeogenesis, activation of AMPK also orchestrates lipid metabolism by directly phosphorylating acetyl-CoA carboxylase (ACC). AMPK activation by metformin phosphorylates and inhibits acetyl-CoA carboxylase (ACC), suppressing *de novo* lipogenesis and reducing hepatic triglyceride accumulation (Xie et al., 2022). *In vivo* murine studies have further demonstrated that AMPK activation suppresses free fatty acid production, reduces hepatic lipid deposition, and promotes fatty acid oxidation via upregulation of lipolytic enzymes such as triglyceride lipase and hormone-sensitive lipase (Lyu et al., 2022). Clinically, these mechanisms translate to improved serum lipid profiles—including modest but consistent increases in HDL cholesterol—and reduced hepatic fat content, supporting its utility in non-alcoholic fatty liver disease (NAFLD) and metabolically unhealthy obesity.

### 3.3 Cardiovascular protection effects

Metformin confers multifaceted cardiovascular protection through hemodynamic, anti-inflammatory, endothelial, and metabolic mechanisms, many of which are AMPK-dependent but extend beyond canonical energy-sensing pathways. A network meta-analysis encompassing 424 randomized controlled trials concluded that metformin exerts a significant antihypertensive effect, although its magnitude is less pronounced than that observed with glucagon-like peptide-1 (GLP-1) receptor agonists or sodium–glucose cotransporter 2 (SGLT-2) inhibitors (Tsapas et al., 2021). Beyond hemodynamic effects, metformin exerts anti-inflammatory actions via AMPK activation, which inhibits nuclear factor-κB (NF-κB)-mediated inflammatory signaling. This results in decreased production of pro-inflammatory cytokines such as tumor necrosis factor-alpha (TNF-α) and interleukin-6 (IL-6), while promoting macrophage polarization toward the anti-inflammatory M2 phenotype. Consequently, metformin improves macrophage dysfunction and reduces foam cell formation (Feng et al., 2021).

The mechanistic synergy between metformin and newer cardiometabolic agents further supports rational combination strategies. For instance, while SGLT2 inhibitors primarily reduce cardiac preload and improve renal sodium handling, and GLP-1 receptor agonists enhance insulin secretion and suppress appetite, metformin uniquely targets hepatic energy metabolism and systemic inflammation—suggesting complementary rather than redundant actions. Ongoing trials are evaluating whether such combinations yield additive or synergistic benefits on hard cardiovascular endpoints.

### 3.4 Effects on aging and longevity

Metformin has emerged as a leading pharmacological candidate for targeting fundamental aging processes—a concept supported by preclinical evidence and now under rigorous clinical evaluation. As a modulator of evolutionarily conserved nutrient-sensing pathways, metformin influences multiple hallmarks of aging, including mitochondrial homeostasis, cellular senescence, stem cell exhaustion, chronic low-grade inflammation (“inflammaging”), and dysregulated intercellular communication (Lopez-Otin et al., 2023). Its ability to activate AMPK and inhibit mTOR signaling converges on key regulators of proteostasis, autophagy, and redox balance—processes whose decline underpins age-related functional deterioration. The landmark Targeting Aging with Metformin (TAME) trial—a multicenter, randomized, placebo-controlled study enrolling over 3,000 adults aged 65–79 years—is designed to determine whether metformin delays the onset or progression of age-associated conditions (e.g., myocardial infarction, stroke, dementia, cancer, frailty) (Kulkarni et al., 2020). Importantly, TAME uses clinical composite endpoints—not surrogate biomarkers—thereby prioritizing real-world health outcomes. If successful, metformin would become the first drug formally evaluated and approved for aging-related indications, shifting the paradigm from disease-specific treatment to healthspan extension—the period of life spent in good health, free from major chronic morbidity.

Taken together, metformin exerts multiple beneficial effects on metabolic regulation, cardiovascular protection, and healthy lifespan extension, positioning it as a promising agent for applications beyond diabetes management. Nevertheless, the clinical use of any therapeutic agent necessitates a careful weighing of benefits against potential risks. In addition to the aforementioned positive effects, metformin is associated with adverse impacts on several physiological systems, including the nervous, digestive, reproductive, and hematologic systems. Moreover, its effects on tumors and neurodegenerative diseases remain subjects of ongoing debate.

## 4 Adverse physiological effects of metformin

Metformin is associated with a range of adverse physiological effects, involving the gastrointestinal and nervous systems, reproductive function, body weight regulation, tumor biology, and acid-base homeostasis. Consequently, clinical management of these potential complications warrants careful evaluation and individualized therapeutic strategies (Table 1).

**TABLE 1:** Treatment strategies for special cases.

Special cases	Treatment strategy	References
Vitamin B12 deficency	①Therapeutic supplements ②Injections of vitamin B12 ③Vitamin B12-rich diet	Khattab et al. (2023)
Acidosis	Hemodialysis	Correia and Horowitz (2022)
Pregnancy	Insulin is recommended and metformin can be used during the first 3 months of pregnancy, with strict monitoring of drug concentration	Abolhassani et al. (2023)
Gastrointestinal adverse reactions	Metformin XR	Nabrdalik et al. (2024)
Renal insufficiency	eGFR≥30 ml ·min^-1^·(1.73 m^2^)^−1^	Chinese Diabetes Society and Guo (2025)

### 4.1 Vitamin B12 deficiency

Patients with T2DM receiving metformin commonly experience symptoms of vitamin B12 deficiency, and this relationship has been established (Table 2). Cross-sectional studies conducted in different regions have reported an association between metformin use and vitamin B12 deficiency in patients with T2DM. Owing to differences in diagnostic criteria, dietary habits, and study populations, the reported prevalence of vitamin B12 deficiency varies considerably across regions, ranging from 14% to 51%. Furthermore, two cross-sectional studies of patients with T2DM taking metformin have demonstrated that long-term (>2 years), high-dose metformin therapy in patients with T2DM can significantly increase the risk of vitamin B12 deficiency, with female patients being at a higher risk (Asghar et al., 2024; Huynh et al., 2024).

**TABLE 2:** Distinguishing between established and unestablished clinical effects under physiological conditions.

Physiological process	Established mechanisms	Possible or unproven mechanisms
Vitamin B12 deficency	Metformin inhibits the absorption of vitamin B12-intrinsic factor complex by antagonizing calcium ions	Current research lacks evidence from randomized controlled trials to confirm this view, and it also cannot confirm whether the cause of vitamin B12 deficiency is the primary disease or metformin use
Neurological dysfunction	Low and high concentrations of metformin can activate AMPK via the mTORC1, PEN2, and AXIN-LKB1 pathways, respectively	Metformin-induced protein inhibition may affect the function of proteins in the nervous system, which requires further experimental confirmation
Acidosis	Metformin causes increased lactate levels by inhibiting the mitochondrial respiratory chain	There is currently no evidence that low concentrations, i.e., clinically therapeutic concentrations, of metformin cause acidosis. (Correia and Horowitz, 2022)
Weight loss	Metformin can help with weight loss by activating Lac-Phe	The effectiveness of weight loss treatment is greatly affected by patient adherence, and its efficacy is uncertain in children, adolescents, and women with POS
Pregnancy	Metformin crosses the placental barrier and inhibits OCT3, which affects serotonin function	Metformin use increases the risk of small-for-gestational-age infants and inhibits neonatal social development, but research on this topic has certain limitations
Gastrointestinal adverse reactions	Metformin can lead to an increase in *Escherichia coli* and *Shigella* in the gut, while inhibiting proteases and enterokinases	The effects of blood glucose fluctuations and long-term metformin use on gut microbiota are unclear

#### 4.1.1 Mechanisms of impaired vitamin B12 absorption

**FIGURE 2:**
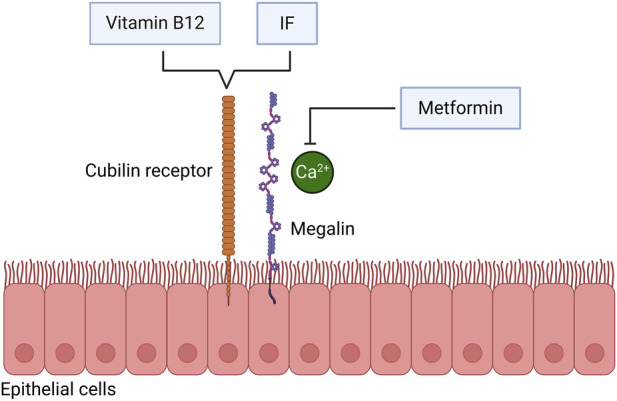
Metformin inhibits the calcium-mediated absorption of vitamin B12. Metformin inhibits the binding of the vitamin B12-intrinsic factor complex to cubilin (CUBN) receptors through its antagonistic effect on calcium ions. IF, intrinsic factor.

Metformin-induced vitamin B12 malabsorption is primarily mediated by calcium antagonism at the cubilin (CUBN) receptor (Figure 2). CUBN is a multi-ligand receptor located mainly in the terminal ileum and responsible for the uptake of the vitamin B12-intrinsic factor (vitamin B12-IF) complex. The Gp280/IF-vitamin B12 receptor, also known as CUBN, is a large endocytic receptor that plays a central role in vitamin B12 homeostasis, as well as in the renal reabsorption of proteins and certain toxic substances, including albumin, vitamin D-binding protein, and cadmium (Kozyraki and Cases, 2020).

Under physiological conditions, vitamin B12 binds to IF secreted by gastric parietal cells to form a stable vitamin B12-IF complex. In the terminal ileum, this complex binds to the CUBN receptor via a calcium-dependent mechanism, which subsequently stabilizes the interaction between CUBN and the vitamin B12-IF complex. Metformin exhibits antagonistic effects on calcium ions; therefore, it can interfere with the binding of the vitamin B12-IF complex to the CUBN receptor. Bauman et al. proposed that protonated metformin molecules interact with the hydrophobic core of the ileal cell membrane, displacing divalent calcium ions by increasing the membrane surface charge. This effect can be reversed by increased calcium intake, providing strong support for this mechanism (Al Zoubi et al., 2024).

Factors such as alterations in the gut microbiota, changes in the enterohepatic circulation of vitamin B12, and reduced IF secretion may also impair vitamin B12 absorption. Nevertheless, the calcium-antagonistic effect of metformin, which causes inhibition of the calcium-dependent uptake of the vitamin B12-IF complex in the terminal ileum, is increasingly being considered as the most plausible mechanism underlying metformin-induced vitamin B12 deficiency. This impairment can be alleviated by calcium supplementation (Bauman et al., 2000; Tiwari et al., 2023).

In a crossover pilot study, Muralidharan et al. (2024) used [13C]-cyanocobalamin as a tracer to quantify the effects of metformin alone and in combination with calcium on vitamin B12 bioavailability. The aims of this study were to confirm the role of calcium in counteracting metformin-induced vitamin B12 malabsorption and determine whether calcium supplementation could reverse the inhibitory effects of metformin. The results revealed that calcium supplementation significantly increased vitamin B12 bioavailability in healthy adults receiving metformin, thereby providing partial support for this mechanism (Muralidharan et al., 2024).

#### 4.1.2 Neurological consequences of B12 deficiency: myelin disruption

Chronic B12 deficiency impairs peripheral and central nervous system function primarily through disruption of myelin synthesis and maintenance. Myelination is an energetically demanding process requiring coordinated fatty acid elongation, phospholipid assembly, and cholesterol biosynthesis—all dependent on B12 as a cofactor for methylmalonyl-CoA mutase (MUT). MUT converts methylmalonyl-CoA to succinyl-CoA, a critical step linking odd-chain fatty acid oxidation and branched-chain amino acid catabolism to the tricarboxylic acid (TCA) cycle. B12 deficiency causes methylmalonic acid (MMA) accumulation, which inhibits mitochondrial fatty acid oxidation and depletes acetyl-CoA pools required for lipid synthesis (Poitelon et al., 2020). Concurrently, elevated MMA incorporates aberrantly into fatty acids, generating neurotoxic methyl-branched lipids that destabilize myelin membranes. As saltatory conduction relies on intact, lipid-rich myelin sheaths to insulate axons and accelerate action potential propagation, structural compromise leads to slowed nerve conduction velocity, sensory neuropathy, and, in severe cases, subacute combined degeneration of the spinal cord (Baba, 2022).

#### 4.1.3 S-adenosylmethionine (SAM) dysfunction associated with vitamin B12 deficiency

Vitamin B12 deficiency results in S-adenosylmethionine depletion, consequently impairing neurotransmitter synthesis and DNA methylation. As a cofactor for methionine synthase, vitamin B12 is required for the conversion of homocysteine to methionine. Methionine is subsequently converted to SAM, a key methyl donor involved in the methylation of several neurotransmitters (e.g., dopamine and serotonin) (Gueant et al., 2013). Vitamin B12 deficiency reduces SAM availability, thereby impairing neurotransmitter synthesis and function. Because SAM is central to cellular methylation reactions, disruption of methionine synthase activity has broad effects on methylation-dependent processes, including epigenetic regulation (Froese et al., 2019). DNA methylation, a major epigenetic mechanism, regulates gene expression by adding methyl groups to cytosine residues and contributes to genomic stability. Consequently, vitamin B12 deficiency may also lead to alterations in DNA methylation.

#### 4.1.4 Hematologic manifestations: megaloblastic anemia

The hematological consequences of vitamin B12 deficiency caused by metformin are exemplified by megaloblastic anemia. Vitamin B12 is required for the conversion of 5-methyl-tetrahydrofolate (5-methyl-THF) to THF. In this process, 5-methyl-THF donates its methyl group to vitamin B12 to form THF, while vitamin B12 is converted to methylcobalamin. Next, methylcobalamin transfers the methyl group to homocysteine to generate methionine in a reaction catalyzed by methionine synthase, with vitamin B12 acting as a coenzyme. In case of vitamin B12 deficiency, methionine production decreases, leading to the so-called methylfolate trap. This phenomenon reflects a physiological response to reduced methyl-group availability resulting from insufficient methionine synthesis. Consequently, the intracellular levels of SAM decline, impairing cellular methylation reactions as described above (Scott and Weir, 1981).

Reduced levels of folate or vitamin B12 are among the most reliable indicators of megaloblastic anemia (Castle, 1978). Deficiency of either vitamin B12 or folate impairs DNA synthesis and can lead to the development of megaloblastic anemia. Candelario and Klein (2022) reported the case of a 75-year-old patient with vitamin B12 levels of 93 pg/mL, markedly below the normal range of 220–600 pg/mL. Peripheral blood smear findings showed macrocytosis, thrombocytopenia, and hypersegmented neutrophils, which are characteristic features of megaloblastic anemia. This evidence further illustrated the association between vitamin B12 deficiency and this condition (Candelario and Klein, 2022).

In summary, metformin inhibits vitamin B12 absorption through calcium antagonism, leading to impaired myelin and neurotransmitter synthesis, disrupted DNA methylation, and the development of megaloblastic anemia. These findings support the need for regular monitoring of vitamin B12 levels and consideration of calcium and vitamin B12 supplementation in patients receiving long-term metformin therapy.

### 4.2 Impact on cognitive function

#### 4.2.1 Metformin modulates neuronal protein homeostasis through AMPK–mTOR signaling

**FIGURE 3:**
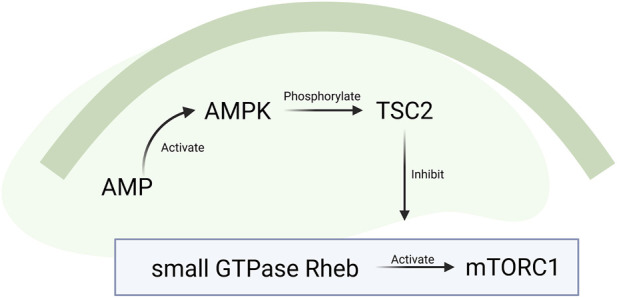
Activation of AMP-activated protein kinase (AMPK) inhibits mammalian target of rapamycin (mTOR). Upon activation, AMPK and AMPK phosphorylate tuberous sclerosis complex 2 (TSC2), thereby inhibiting mTOR complex 1 (mTORC1) activation by the small GTPase Ras homolog enriched in brain (Rheb).

Metformin may influence cognitive function (Table 3), with a proposed mechanism involving its effects on the nervous system, including inhibition of protein synthesis. The pharmacological effects of metformin, particularly the activation of AMPK and suppression of mammalian target of rapamycin (mTOR) signaling, are widely acknowledged (Figure 3) (Bharath and Nikolajczyk, 2021). These effects inhibit protein synthesis, thus potentially restricting cellular growth and disrupting energy metabolism in neural cells. In mammalian cells, growth and proliferation rely on adequate nutrient supply to support biosynthesis and a sufficient energy source, with mTOR playing a key regulatory role (Hardie, 2008).

**TABLE 3:** Summary of pathways affecting cognitive function.

Pathway type	Pathway mechanism	References
Inhibiting protein synthesis	mTOR phosphorylates 4E-BP1, releasing eIF4E to initiate mRNA translation and protein synthesis	Wang and Jia (2023)
	Metformin activates AMPK, which inhibits mTOR by phosphorylating TSC2 and RAPTOR.	(Yang et al., 2017; Bharath and Nikolajczyk, 2021)
Affecting neurotransmitter function	Metformin inhibits ATP hydrolysis by affecting respiratory chain complex I, thereby inhibiting the function of clathrin and SNARE proteins and hindering cell membrane fusion and neurotransmitter endocytosis	(Baschieri et al., 2020; Chanaday and Kavalali, 2021; Kim et al., 2021; Xian et al., 2021; Smith and Smith, 2022; Jahn et al., 2024)

AMPK phosphorylates tuberous sclerosis complex 2 (TSC2), thereby increasing its activity. This phosphorylation is essential for regulating translation and controlling cell size during energy stress (Inoki et al., 2003). At the cellular level, tuberous sclerosis is linked to abnormal mTOR signaling (Dhamne et al., 2023). The mTOR complex 1 (mTORC1) is activated by the small GTPase Ras homolog enriched in brain (Rheb) and inhibited by proline-rich Akt substrate, 40 kDa (PRAS40) (Yang et al., 2017). TSC2 acts as a negative regulator of mTORC1 by inhibiting Rheb activity, which decreases the interaction between Rheb and mTORC1 and suppresses mTORC1 signaling. Howell et al. demonstrated that low doses of metformin (≤100 mg/kg) activate the TSC complex, leading to inhibition of Rheb-GTP and subsequent blockade of mTORC1. In cells lacking TSC1, mTORC1 remains constitutively active, and the level of protein synthesis inhibition decreases from 75% to 25%, confirming that mTORC1 is the main target of this pathway (Howell et al., 2017).

Gwinn et al. further demonstrated that AMPK directly phosphorylates raptor, an mTOR-binding partner, at two conserved serine residues. This phosphorylation is essential for effective inhibition of mTORC1 (Gwinn et al., 2008). Accordingly, activation of AMPK and suppression of mTORC1 signaling via AMPK-mediated phosphorylation of RAPTOR at Ser722 and Ser792, as well as TSC2 at Ser1387, have been reported (Van Nostrand et al., 2020). Under glucose deprivation, AMPK also promotes autophagy by directly phosphorylating unc-51 like autophagy activating kinase 1 (Ulk1) at Ser317 and Ser777 (Kim et al., 2011). Nonetheless, some studies suggested that although ULK1-mediated inhibition of mTORC1 decreases protein synthesis, the induction of autophagy helps clear damaged mitochondria, invading pathogens, and toxic protein aggregates. This process may assist in preventing and treating AD (Wang and Jia, 2023; Pareek and Kundu, 2024).

Excessive phosphorylation of AMPK suppresses mTOR and S6K1 activity, inhibits protein synthesis, and causes leucine resistance, thereby decreasing leucine-stimulated protein synthesis (Shi et al., 2020). Under normal conditions, mTOR phosphorylates 4E-binding protein 1 (4E-BP1), rendering it inactive and freeing eukaryotic translation initiation factor 4E (EIF4E) to initiate cap-dependent mRNA translation (Takahashi et al., 2022). By inhibiting mTOR signaling, AMPK ultimately reduces protein synthesis by preventing the initiation of mRNA translation.

#### 4.2.2 Reduced protein synthesis affects neurotransmitter function

Activation of AMPK inhibits protein synthesis, thereby impacting the production and release of neurotransmitters. Neuropeptides also play a crucial regulatory role in the nervous system and work concertedly with classical neurotransmitters (Neugebauer et al., 2020). This process further affects proteins involved in membrane fusion, including syntaxin and synaptobrevin (Chanaday and Kavalali, 2021; Fu et al., 2023). Soluble NSF attachment protein receptor (SNARE) proteins form functional complexes via specific protein–protein interactions that facilitate membrane fusion. In addition to potentially reducing SNARE protein synthesis via AMPK activation, metformin may directly inhibit SNARE protein activation. As AMPK inhibits protein synthesis and high doses of metformin decrease ATP production, this mechanism reduces the synthesis and impairs the functions of SNARE proteins.

The reuse of SNARE proteins across successive membrane fusion events depends on the rapid disassembly of SNARE complexes after fusion. This disassembly is aided by the ATPase NSF, allowing further rounds of membrane fusion (Jahn et al., 2024). Thus, SNARE activation and recycling rely heavily on ATP hydrolysis (Kim et al., 2021). Metformin interacts with mitochondrial complex I and other components of the electron transport chain, thereby inhibiting oxidative phosphorylation and decreasing ATP production (Xian et al., 2021).

Clathrin-mediated endocytosis enables cells to selectively internalize molecules based on changing cellular needs (Smith and Smith, 2022). In addition, it is the major pathway for the uptake of most cell-surface receptors and their ligands, including neurotransmitters (Baschieri et al., 2020). The release of clathrin from endocytic vesicles depends on the phosphorylation of Hsc70, for which an adequate ATP reserve is required (Seike et al., 2024; Sengul et al., 2024). By decreasing ATP synthesis, metformin prevents the detachment of clathrin from endocytic vesicles, thus blocking neurotransmitter endocytosis. AMPK-induced autophagy can facilitate the clearance of harmful substances in the nervous system to a certain extent. Nevertheless, excessive autophagy may amplify inflammation and promote atherosclerosis by triggering the release of inflammatory cytokines and damage-associated molecular patterns (Bu et al., 2023; Iba et al., 2024).

Although these inhibitory effects may manifest systemically, the high energy demands of neurons render them particularly vulnerable to metformin-induced suppression of protein synthesis. Collectively, metformin-mediated inhibition of protein synthesis poses a potential threat to neuronal growth, metabolic homeostasis, and neurotransmitter signaling. These findings highlight the need for clinical vigilance regarding its potential impact on nervous system function, as well as further research to establish the causal relationship.

### 4.3 Metformin-associated lactic acidosis

As discussed above, metformin inhibits the mitochondrial respiratory chain, which may lead to increased lactate production. Metformin-associated lactic acidosis is a rare but potentially life-threatening side effect (Correia and Horowitz, 2022). When plasma metformin levels are >5 mg/L, the risk of lactic acidosis increases significantly (Lalau et al., 2011). As metformin is primarily eliminated by the kidneys, it may accumulate in patients with impaired renal function, exacerbating the risk of lactic acidosis (Rahman and Tuba, 2022).

In case a patient receiving metformin develops lactic acidosis, metformin-associated lactic acidosis should be considered, along with sepsis, when serum creatinine levels are ≥256 μmol/L and lactate levels are ≥8.4 mmol/L (van Berlo-van de Laar et al., 2020). Emerging evidence suggests that renal replacement therapy, rather than alkali supplementation alone, is the preferred therapeutic modality (Moioli et al., 2016). In addition, metformin is contraindicated in conditions associated with tissue hypoxia, including liver failure and poorly controlled diabetes (Di Mauro et al., 2022).

### 4.4 Modulators of metformin-induced weight change

In addition to its proven glucose-lowering effects, metformin offers a modest weight-loss benefit (Xiao et al., 2024a). However, multiple factors can significantly influence this effect. As of 2023, obesity is a major global health concern that affects approximately 2 billion individuals worldwide, primarily due to a sedentary lifestyle and unhealthy dietary patterns (Zhang et al., 2023). It has been shown that lactoyl-phenylalanine promotes weight loss by suppressing appetite, with lactate being its key precursor. As metformin increases lactate levels, it has been proposed to induce weight loss via lactoyl-phenylalanine activation; this hypothesis is supported by Scott et al. (2024).

The Diabetes Prevention Program is the largest study evaluating the effect of metformin on body weight. This study demonstrated that metformin could lower body weight; however, the extent of this effect depends largely on patient adherence to treatment. Consequently, the weight-loss benefits of metformin vary significantly across individuals (Yerevanian and Soukas, 2019). Consistent with this observation, experiments showed significant variability among the participants in their weight-loss response to metformin (Yerevanian and Soukas, 2019).

Furthermore, the weight-loss effect of metformin is modulated by patient-specific factors, including age and disease status. The effectiveness of metformin for weight loss in children and teenagers remains uncertain. Masarwa et al. evaluated the safety and effectiveness of metformin compared with placebo when combined with lifestyle changes in children and teenagers with obesity. The randomized controlled trials included in this analysis exhibited significant heterogeneity, suggesting uncertainty regarding the effectiveness of metformin in this population (Masarwa et al., 2021). Certain diseases may also attenuate the weight-loss effect of metformin.

Metformin appears to have limited weight-lowering effects in women with polycystic ovary syndrome (Ziqubu et al., 2023). A randomized controlled trial involving women with polycystic ovary syndrome (PCOS) demonstrated that short-term (60 days) administration of metformin (1,500 mg/day) did not significantly alter brown adipose tissue (BAT) activity or plasma irisin levels, indicating a lack of effect on thermogenesis-related energy expenditure. These findings suggest that, in this specific population, metformin does not exert a meaningful weight-reducing effect through BAT activation. Furthermore, short-term treatment alone appears insufficient to induce significant weight loss (Oliveira et al., 2020).

The human microbiome and genetic factors influence obesity and must be considered when prescribing metformin. For instance, individuals with obesity tend to exhibit a higher Firmicutes-to-Bacteroidetes ratio in their gut microbiota than those with normal weight (Magne et al., 2020). Regarding genetic susceptibility, genome-wide association studies have identified that the fat mass and obesity-associated gene plays a key role in human obesity (Chauhdary et al., 2021).

The weight-loss effect of metformin is mechanistically attributed to its promotion of lactoyl-phenylalanine synthesis, which suppresses appetite. However, its clinical efficacy is modulated by multiple factors—including patient adherence, age, diseases, gut microbiota composition, and genetic background—precluding a definitive conclusion regarding its overall weight-loss benefit.

### 4.5 Reproductive and developmental implications

Metformin crosses the placental barrier, posing potential risks to fetal nervous system development, growth, and social function. Definitive evidence for fetal harm associated with exposure to metformin is not available; however, current data are inadequate to rule out potential risks. Hence, the drug dosage should be carefully regulated during pregnancy (American Diabetes, 2021; Torunoglu et al., 2023). Metformin can inhibit OCT3 (Vachalova et al., 2024) and, in humans, serotonin transport across the placenta is facilitated by OCT3/solute carrier family 22 member 3 (OCT3/SLC22A3) (Karahoda et al., 2020). Thus, serotonin homeostasis may be disrupted following inhibition of this transporter by metformin. Serotonin is crucial for the development of the fetal nervous system, particularly brain maturation. Moreover, this neurotransmitter is involved in regulating emotion, behavior, and cognition (Hanswijk et al., 2020; Karmakar and Lal, 2021), and plays a prominent role in the placenta–brain axis (Vachalova et al., 2024).

Therefore, exposure to metformin during pregnancy can potentially affect fetal development (Shah et al., 2018). A study indicated that metformin may aggravate the risk of small-for-gestational-age outcomes (defined as birth weight below the 10th percentile) by approximately 20%–30%, possibly by inhibiting mitochondrial function and interfering with one-carbon metabolism (Owen et al., 2021). Furthermore, another study revealed that prenatal exposure to metformin or selective loss of OCT3 impairs social interaction preference in male mice. This effect may be associated with abnormal OCT3-mediated placental transport of serotonin and tryptophan. Alternatively, it could be linked to metformin-induced AMPK activation, which inhibits protein synthesis and consequently affects synaptic development (Garbarino et al., 2019).

### 4.6 Gastrointestinal adverse effects

The adverse effects of metformin on the digestive system primarily manifest as indirect consequences of gut microbiota alteration, alongside direct impacts on digestive and absorptive functions. The lipopolysaccharides released by *Escherichia coli* and *Shigella* can cause gastrointestinal side effects, such as bloating and diarrhea (Chan et al., 2024). In prospective cohort studies, Gao et al. found that metformin can lower microbial diversity, thereby disrupting the balance of the intestinal microbiota. Furthermore, the drug may reduce beneficial bacterial genera, such as *Romboutsia*, or increase the abundance of opportunistic pathogens, leading to gastrointestinal discomfort. Similarly, another study reported that metformin inhibits enterokinase and trypsin, thereby impairing intestinal protein digestion and vitamin B12 absorption. This mechanism is intricately associated with the gastrointestinal side effects, weight loss, and vitamin deficiency noted in clinical practice (Gao et al., 2024).

## 5 Impact of metformin on human pathophysiology

### 5.1 Anticancer potential and controversies of metformin

#### 5.1.1 Canonical AMPK–mTOR–mediated antitumor mechanisms

Metformin, the first-line therapeutic drug for T2DM, has attracted immense attention owing to its potential anti-tumor activity. Experimental evidence has consistently shown that metformin prevents tumor cell proliferation, induces apoptosis, and arrests the cell cycle. These effects are predominantly facilitated by the activation of AMPK, which subsequently inhibits the mTOR signaling pathway, a key regulator of cell growth, proliferation, and metabolism (Sinnett-Smith et al., 2013; Jahani and Davoodi, 2022). A crucial aspect of this mechanism is the suppression of mTORC1. Collectively, these observations highlight the central role of AMPK-mediated mTORC1 inhibition in the anti-proliferative activity of metformin.

Building on the central role of AMPK-mediated mTORC1 inhibition, metformin exerts further anti-tumor effects through coordinated regulation of translation and cell cycle progression. For instance, metformin increases the concentrations of hypophosphorylated 4E-BPs, which in turn inhibit the assembly of the eukaryotic initiation factor 4F complex; cap-dependent mRNA translation is thus hindered, ultimately inducing apoptosis (Bhat et al., 2016). In addition, metformin can simultaneously inhibit mTORC1 and mTORC2 via the AMPK pathway, inducing G0/G1 cell cycle arrest and apoptosis. Wang et al. (2018) established that this dual inhibition decreases the phosphorylation of 4E-BP1 downstream of mTORC1 and reduces AKT phosphorylation downstream of mTORC2. Consequently, proliferative signaling is diminished, the proportion of cells in the G0/G1 phase is increased, and the formation of autophagosomes is enhanced (Wang et al., 2018). These mechanistic insights demonstrate the multifaceted nature of metformin’s anti-tumor activity, encompassing both translational control and cell cycle regulation.

These findings are further reinforced by research conducted by Fan et al. (2021), which showed that AMPK activation inhibits the proliferation of canine mammary tumor cells by coordinating the suppression of the mTOR/Akt pathway, regulating cyclins to cause G0/G1 arrest, and activating the mitochondrial apoptotic pathway. Taken together, these studies provide a coherent mechanistic framework, supporting the conclusion that metformin primarily exerts its anti-tumor effects through AMPK activation and subsequent inhibition of mTOR signaling.

#### 5.1.2 Contextual duality of downstream effectors: the 4E-BP1 paradox

Conversely, an increasing body of evidence suggests that the role of key downstream effectors, such as 4E-BP1, in cancer is complex and highly context-dependent. Moreover, intracellular signaling states and the tumor microenvironment influence the process. Although 4E-BP1 functions as a tumor suppressor in the canonical AMPK/mTOR pathway, it can also enable tumor cells to adapt to metabolic and genotoxic stress by selectively regulating mRNA translation, thereby exhibiting oncogenic properties. Owing to this selective regulation, tumor survival and progression may be supported in adverse conditions.

**FIGURE 4:**
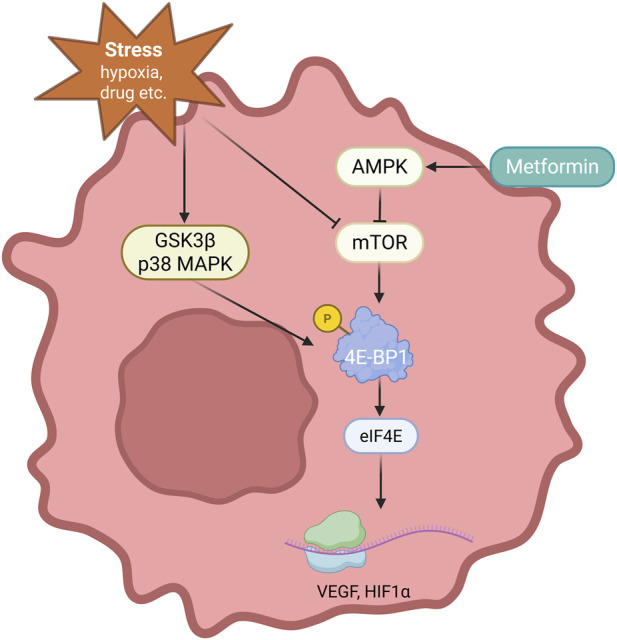
Mechanism of action of insulin and 4E-binding protein 1 (4E-BP1) on tumor cell survival. Metformin can inhibit mTOR while activating 4E-BP1, thereby promoting the synthesis of vascular endothelial growth factor (VEGF), hypoxia inducible factor 1 subunit alpha (HIF1α), and other substances. This promotes tumor formation and maintenance. GSK3β, glycogen synthase kinase 3 beta; MAPK, mitogen-activated protein kinase.

The functional outcome of 4E-BP1 is further modulated by stress-responsive kinases that bypass canonical mTOR signaling. Under stressful conditions (e.g., hypoxia, viral infection, or ultraviolet exposure) 4E-BP1 can be phosphorylated by kinases independent of mTOR, including p38 mitogen-activated protein kinase (MAPK) and extracellular signal-regulated kinase (ERK), which relieves its translational repression. For example, phosphorylated 4E-BP1 can facilitate the cap-independent translation of specific mRNAs encoding survival-related factors, such as vascular endothelial growth factor (VEGF) and hypoxia inducible factor 1 subunit alpha (HIF1α), thereby promoting tumor angiogenesis and adaptation to hypoxic environments (Qin et al., 2016). This context-specific reprogramming of protein synthesis can aid 4E-BP1 in paradoxically supporting tumor growth and enhancing stress tolerance (Figure 4).

Therefore, the overall molecular effects of metformin, which mainly influence 4E-BP1 phosphorylation via mTOR inhibition, may differ depending on the signaling environment within a specific tumor. The regulation of 4E-BP1 remains mostly theoretical, supported primarily by experimental data, with no confirmed clinical validation to date. This functional duality highlights the need to interpret the effects of metformin beyond a simple linear pathway and underscores the importance of further research to understand how these competing mechanisms affect its therapeutic potential. In summary, the therapeutic impact of metformin must be evaluated in the context of 4E-BP1’s dynamic and context-specific roles, emphasizing the necessity for integrative mechanistic studies.

#### 5.1.3 Clinical evidence: discrepancies between epidemiology and interventional trials

Collective evidence from epidemiological and clinical studies suggests that metformin offers an overall anticancer benefit, especially in patients with diabetes. However, its efficacy differs significantly based on the context. A recent meta-analysis observed a significant decrease in overall cancer incidence among metformin users (pooled risk ratio: 0.78, 95% confidence interval [CI]: 0.70–0.87), asserting its potential chemopreventive role. This benefit is most apparent in diabetic populations, where indirect effects (e.g., improved hyperinsulinemia and reduced systemic inflammation) likely work concertedly with direct modulation of the AMPK/mTOR pathway (O’Connor et al., 2024). However, other randomized controlled trials (Home et al., 2010) and meta-analyses (Stevens et al., 2012) failed to confirm that metformin consistently exerts an independent anticancer effect, emphasizing the differences between observational and interventional evidence. In contrast, randomized controlled trials in patients with cancer who do not have diabetes have reported neutral results. The MA.32 trial (Goodwin et al., 2022) did not find an improvement in invasive disease-free survival with adjuvant metformin in early-stage breast cancer (hazard ratio: 1.01, 95% CI: 0.84–1.21). Similarly, a phase II study on advanced pancreatic cancer (Kordes et al., 2015) failed to demonstrate a survival benefit (overall survival at 6 months: 63.9% vs. 56.7% months (Placebo group vs. metformin group), respectively, *p* = 0.41). Hence, direct antitumor effects of the drug are likely limited to specific biological settings and may not be broadly applicable.

Cancer type-specific analyses further establish this heterogeneity. Meta-analyses of randomized controlled trials in breast cancer demonstrated improvements in metabolic markers, such as decreased insulin resistance, and positive survival trends in diabetic subgroups. These findings indicate interaction with hormone-related and metabolic factors (Rahmani et al., 2020). In colorectal cancer, Meng et al. (2017) observed a survival benefit in patients with diabetes (hazard ratio: 0.56, 95% CI: 0.39–0.80). In contrast, Ng et al. (2020) did not identify an overall survival advantage in more inclusive populations, suggesting confounding by metabolic comorbidities. Moreover, mixed results have been reported for hepatocellular carcinoma. Cunha et al. (2020) observed a reduced risk in patients with diabetes who were treated with metformin (odds ratio: 0.5, 95% CI: 0.4–0.7). Xiao et al. (2024b) highlighted that obesity and steatotic liver disease are dominant risk modifiers that potentially obscure the independent contribution of the drug. Furthermore, a meta-analysis on prostate cancer (He et al., 2019) documented only modest reductions in biochemical recurrence (risk ratio: 0.87, 95% CI: 0.76–1.00). These inconsistencies could be attributed to variations in tumor biology, metabolic status, and pathway dependence among cancer types.

These clinical and epidemiological data collectively highlight the context-dependent nature of the anticancer effects of metformin. The benefits are consistent in diabetic populations and could be explained by systemic metabolic improvements and direct modulation of intracellular signaling pathways. Broad clinical generalizations are difficult owing to variability across cancer types, patient metabolic status, and study design. Therefore, further investigations should prioritize stratified clinical trials that consider tumor molecular characteristics, metabolic profiles, and pathway activation status. Such studies may aid in identifying responsive subgroups and establishing clear indications for the use of metformin in cancer prevention and treatment.

### 5.2 Impact on neurodegenerative diseases

The therapeutic effects of metformin on neurodegenerative diseases, particularly AD and Parkinson’s disease (PD), are complex and context dependent, and studies have reported both neuroprotective and detrimental effects (Table 4). Various factors, including the disease model, genetic background, dosage, and treatment duration, influence this duality.

**TABLE 4:** Research summary and pattern analysis of metformin effects on neurodegenerative diseases.

Disease	Model	Metformin treatment	Primary outcome	Key findings and proposed mechanism	Ref.
Alzheimer’s disease (AD)	Mouse	100 mg/kg, 17 days	Beneficial	Improved cognition, reduced Aβ and p-tau. Mechanism: AMPK activation, mTOR inhibition	Ale Mahmoud Mehraban et al. (2024)
	ApoE^−/−^ mouse	300 mg/kg/day, 18 weeks	Detrimental	Failed AMPK activation, ∼2x increase in p-tau, neuronal loss. Linked to elevated triglycerides	Kuhla et al. (2019)
	Astrocyte *in vitro*	2.5 mM, 48 h	Complex/Null	Activated AMPK but did not reduce Aβ secretion. AMPK inhibition decreased Aβ levels	Garcia-Juan et al. (2024)
Parkinson’s disease (PD)	Clinical, low-dose​	<300 cDDD <10 DDD/month	Beneficial	Associated with reduced risk of Parkinson’s disease	Huang et al. (2022)
	Clinical, high-dose	≥300 cDDD, ≥10 DDD/month	Null	No significant neuroprotective effect observed	Huang et al. (2022)
	Mouse, post-injury	200 mg/kg, 7 days (post-MPTP)	Beneficial	lowers α-synuclein phosphorylation and protected dopaminergic neurons	Katila et al. (2017)
	Mouse, co-injury​	150 mg/kg, 7 days (4 times a day with MPTP)	Detrimental	Inhibition of microglia activation and aggravation of dopaminergic neuron damage	Ismaiel et al. (2016)

#### 5.2.1 Impact on AD

Accumulating preclinical evidence suggests that metformin exerts multifaceted effects on Alzheimer’s disease (AD) pathology through metabolic and signaling modulation. In animal models of AD, metformin has demonstrated neuroprotective effects, including improvements in cognitive function, reductions in Aβ deposition, regulation of tau phosphorylation, and attenuation of neuroinflammation. Mechanistically, the drug modulates Aβ-related enzymes as well as kinases and phosphatases involved in tau phosphorylation by activating AMPK and inhibiting mTOR signaling (Khezri et al., 2022). A study found that oral administration of metformin at 100 mg/kg for 17 days significantly improved spatial learning, memory, and passive avoidance behavior in rats. The coadministration of the AMPK inhibitor dorsomorphin and the mTOR activator MHY1485 abolished these effects, asserting that metformin exerts its effects by activating AMPK and inhibiting mTOR (Ale Mahmoud Mehraban et al., 2024).

Despite these promising findings, accumulating evidence also points to significant heterogeneity and context dependence in the effects of metformin on AD-related pathology. The effects of metformin vary by cell type, dose, treatment duration, and individual, particularly with respect to Aβ secretion. García-Juan et al. observed that metformin did not enhance autophagy or reduce Aβ secretion, although it activated AMPK in astrocytes. In contrast, inhibition of AMPK decreased Aβ levels, suggesting that the neuroprotective effects of the drug are not universal (Garcia-Juan et al., 2024). Similarly, Kuhla et al. (2019) found that metformin failed to activate AMPK in apolipoprotein E−(ApoE)-deficient mice and instead caused a nearly two-fold increase in tau phosphorylation, accompanied by neuronal loss. These effects could be ascribed to increased plasma triglyceride levels, underscoring potential individual risks and cautioning against the indiscriminate use of metformin for managing neurodegeneration.

Taken together, current evidence underscores the complexity of translating metformin’s molecular effects into clinical benefit in AD. Evidence regarding the role of metformin in AD is complex, highlighting the context-dependent nature of its therapeutic effects. Increased tau phosphorylation and neuronal loss are among the adverse outcomes most strongly associated with specific genetic predispositions, particularly lipid-altering mutations in the ApoE gene (Kuhla et al., 2019). Therefore, the potential benefits of metformin depend on the metabolic context of insulin resistance and the absence of specific genetic risk factors, such as the ApoE4 allele. Thus, individualized assessments rather than the indiscriminate use of metformin are needed in clinical practice. Some *in vitro* studies investigating tau modulation in AD have used high drug concentrations and long incubation times, as demonstrated by Gupta et al. (2011). The clinical benefits of metformin in AD are likely due to indirect systemic mechanisms, including broad metabolic improvements, anti-inflammatory effects, and enhanced autophagic function via multiple pathways. Hence, the primary therapeutic value of metformin appears to lie in its ability to modulate the systemic environment in a manner conducive to neuroprotection, rather than in direct, monospecific actions on brain targets.

#### 5.2.2 Impact on PD

The role of metformin in Parkinson’s disease (PD) remains highly debated, reflecting a complex balance between potential neuroprotection and adverse effects. While metformin can activate AMPK and improve mitochondrial function, its long-term use may aggravate disease pathology via vitamin deficiency, mitochondrial inhibition, and inflammatory imbalance. The risk of PD associated with metformin appears to depend on dose, treatment duration, and individual susceptibility, with evidence of a dose–response relationship. Huang et al. (2022) reported that low-dose (<300 cumulative defined daily dose [DDD]) and low-intensity (<10 DDD/month) metformin use were associated with a lower risk of PD, whereas high-dose (≥300 cumulative DDD) and high-intensity (≥10 DDD/month) use showed no neuroprotective benefit. Furthermore, metformin dosage varies among individuals with vitamin B12 deficiency, older adults, and patients with other conditions such as chronic obstructive pulmonary disease (Huang et al., 2022).

Experimental studies further illustrate the context-dependent and sometimes contradictory effects of metformin in PD models. In animal experiments, the effect of metformin on PD model mice remains controversial, potentially due to factors such as the timing of administration, dose, modeling protocol, and animal breed. For instance, in a study, the concomitant administration of 10 mg of 1-methyl-4-phenyl-1,2,3,6-tetrahydropyridine (twice daily) on the first 2 days of treatment with metformin (150 mg/kg four times daily) for 7 consecutive days significantly inhibited microglia activation and worsened dopaminergic neuron damage (Ismaiel et al., 2016). However, in another study, administration of 1-methyl-4-phenyl-1,2,3,6-tetrahydropyridine (30 mg/kg) for 7 days followed by treatment with metformin (200 mg/kg) for 7 days protected dopaminergic neurons and improved behavioral impairment (Katila et al., 2017). The apparent discrepancy between these observations could be explained by the fact that the former is primarily due to acute mitochondrial toxicity, and metformin synergistically inhibits complex I. In contrast, the latter has a long duration, and the anti-inflammatory and pro-autophagy effects of metformin are superior (Katila et al., 2017).

The evidence highlights a specific and context-dependent therapeutic profile for metformin, moving beyond a simple benefit-or-harm view. The role of this drug depends critically on precise dosing and, most importantly, on timing relative to disease development or injury. Clinical evidence suggests that low-intensity use may provide benefits, while higher doses tend to have diminished effects. Mechanistically, this finding supports the idea that timing is essential: treatment after a damaging event may offer protection, possibly by modulating neuroinflammatory glial responses, whereas exposure during acute toxicity could be harmful. Therefore, the neuroprotective potential of metformin in PD relies on optimal timing and dosing, emphasizing the need for tailored treatment strategies to guide future research and clinical practice.

## 6 Limitations of the studies

Despite the widespread clinical use of metformin and its well-established glycemic benefits, several critical limitations across existing studies constrain the understanding of its broader effects. These limitations span methodological inconsistencies, gaps in mechanistic evidence, and challenges in translating preclinical findings to clinical practice.

### 6.1 Methodological constraints in observational studies

A substantial portion of the evidence regarding metformin’s inhibition of vitamin B12 absorption derives from observational studies. These investigations are inherently limited by residual confounding, as extraneous variables such as variations in nutritional status and dietary intake cannot be fully controlled. Consequently, it remains challenging to ascertain whether the typical symptoms of vitamin B12 deficiency are attributable to the medication itself or to the underlying disease state. This ambiguity underscores the need for more rigorously controlled prospective studies to establish causality.

### 6.2 Ambiguities in nervous system and cognitive effects

The implications of metformin for nervous system function and cognition are primarily inferred from indirect mechanistic associations. Given that proteins are integral to nervous system structure and function and given evidence that high concentrations of metformin inhibit protein synthesis (Shi et al., 2020), negative effects on the nervous system have been hypothesized. However, such inferences remain speculative and require direct experimental validation.

Similarly, the potential influence of metformin on neurodegenerative diseases, particularly the AMPK-mediated cognitive benefits observed in certain Alzheimer’s disease (AD) models, is constrained by significant translational gaps. Findings from animal studies, such as those using ApoE-deficient mice, demonstrate high sensitivity to experimental variables including genetic background, dosing regimens, timing of intervention, and strain, all of which complicate extrapolation to human populations. Furthermore, *in vitro* studies investigating tau modulation (e.g., Gupta et al., 2011) frequently employ supraphysiological doses and extended incubation periods that do not reflect clinically safe concentrations (<5 mg/L) or typical treatment durations. The observed cognitive benefits in AD models may also be explained by indirect systemic effects, such as improved metabolic parameters, rather than by direct interactions with amyloid-beta or tau pathology within the central nervous system. Compounding this issue is a general lack of clinical evidence elucidating metformin’s mechanism of action within the central nervous system, as well as the context-dependent nature of its effects, which collectively hinder its therapeutic application. Addressing these gaps will require human-relevant models and biomarker-guided clinical trials.

### 6.3 Safety evidence gaps: lactic acidosis management

While metformin concentrations in the body are normally maintained at micromolar levels insufficient to induce lactic acidosis—a serious but rare side effect—the risk is significantly amplified in patients with impaired renal function who exhibit reduced drug clearance (Rahman and Tuba, 2022). Clinical guidelines therefore restrict metformin use to patients with an estimated glomerular filtration rate (eGFR) of ≥30 mL min^-1^ (1.73 m^2^)^−1^, recommending discontinuation if eGFR falls below this threshold (Chinese Diabetes Society and Guo, 2025). Notably, survival rates following hemodialysis therapy in cases of metformin-associated lactic acidosis have not been systematically assessed, representing an important gap in patient safety data.

### 6.4 Heterogeneity in weight-modulating effects

The effect of metformin on weight loss remains equivocal, partly due to mechanistic and population-specific variability. The appetite-suppressing metabolite lactoyl-phenylalanine has been shown to be strongly correlated with metformin (Spearman’s ρ = 0.76), with the drug increasing its concentration by 150%–200% (Scott et al., 2024). However, contradictory findings exist, with some studies reporting no statistically significant difference in weight loss between metformin-treated subjects and those receiving placebo (Yerevanian and Soukas, 2019). Moreover, investigations into the weight-loss effects of metformin in children and adolescents are constrained by high heterogeneity across study designs and populations (Masarwa et al., 2021). Given the short treatment duration, the exclusion of participants with abnormal glucose metabolism, and the inherent limitations of current adipose tissue detection techniques, the researchers acknowledge that the potential weight-loss effect of metformin in patients with PCOS cannot be entirely excluded (Oliveira et al., 2020). As a result, no definitive conclusion can currently be drawn regarding the efficacy of metformin for weight loss.

### 6.5 Challenges in reproductive system research

Research on metformin’s effects during pregnancy is complicated by several factors. The absence of a gold standard for diagnosing gestational diabetes mellitus introduces potential selection bias in subject recruitment. Furthermore, pregnancy itself is an inherently complex physiological state, making it difficult to control for all extraneous variables across experimental conditions (Owen et al., 2021).

Beyond pregnancy, investigations into metformin’s effects on social behavior remain in their infancy. To date, only mouse model studies have explored this area. These studies are limited by small sample sizes and a lack of standardized, objective criteria for assessing decreased social interaction in animals, rendering the conclusions somewhat arbitrary and subjective (Garbarino et al., 2019). The underlying mechanisms through which metformin might exert such effects warrant further investigation.

### 6.6 Gut microbiota–host interaction uncertainties

Metformin’s impact on the gut microbiota is well recognized; however, disentangling the drug’s direct effects from those related to glycemic status remains a challenge, as both factors influence microbial composition (Chan et al., 2024). Future clinical trials should therefore aim to minimize confounding from variables such as blood glucose levels. More importantly, the long-term association between metformin-induced changes in the gut microbiota and clinical outcomes is largely unknown, representing a critical area for future research (Gao et al., 2024).

### 6.7 Anticancer evidence deficits

Although preclinical studies have suggested potential antitumor effects of metformin, such as 4E-BP1-driven apoptosis via AMPK/mTOR signaling, several key limitations temper these findings. *In vitro* experiments (e.g., Bhat et al., 2016) frequently employ supraphysiological doses (0.1–10 mM) that far exceed clinically relevant concentrations (approximately 0.005 mM). For instance, a dose of 10 mM would likely induce fatal lactic acidosis *in vivo*, thereby compromising the clinical applicability of such results. Furthermore, the proposed oncogenic role of 4E-BP1 under stress conditions such as hypoxia has only been investigated *in vitro*, with no supporting data from human tumor tissues or clinical trials. Additionally, documented clinical benefits, such as reduced cancer incidence, may be attributable to indirect metabolic effects rather than direct antitumor activity, given that confounding factors remain inadequately accounted for. Addressing these limitations will require the development of dose-standardized preclinical models and the execution of biomarker-driven clinical trials.

In summary, despite considerable progress in elucidating the broad pharmacological effects and potential risks of metformin, notable methodological and evidentiary limitations persist. Future research should focus on establishing a causal link between metformin use and the aforementioned adverse effects. Clinically, greater emphasis should be placed on interindividual variability, and treatment efficacy may be optimized through individualized regimens or combination therapies.

## 7 Future prospects

### 7.1 Defining core therapeutic value amid evolving clinical paradigms

Metformin remains the foundational first-line pharmacotherapy for type 2 diabetes mellitus (T2DM), valued for its robust, mechanism-based efficacy in suppressing hepatic gluconeogenesis, enhancing peripheral insulin sensitivity, and maintaining weight neutrality—with a near-zero risk of hypoglycemia (Corcoran and Jacobs, 2023). Long-term use is associated with sustained glycemic control, improved endothelial function, and reduced cardiovascular mortality—effects attributable not only to glucose lowering but also to AMPK-mediated anti-inflammatory, antioxidant, and vasoprotective actions (de Jager et al., 2014). The continued use of this drug as a first-line agent is also based on its established safety record and low cost. However, the clinical use of metformin warrants consideration beyond initiation of treatment. At present, the major limitations are gastrointestinal intolerance, which affects a considerable proportion of patients, and contraindication in individuals with severe renal impairment, owing to the rare but serious risk of lactic acidosis (Flory and Lipska, 2019; Corcoran and Jacobs, 2023). Importantly, while anticancer effects are biologically plausible, high-quality randomized trials consistently demonstrate no survival benefit when metformin is added to standard oncologic regimens—confirming its role as a metabolic adjunct, not a primary antineoplastic agent (Coyle et al., 2016). Likewise, guidelines explicitly advise against metformin monotherapy for weight management, given its modest and highly variable effect size (Liu et al., 2023).

### 7.2 Evidence-driven therapeutic reassessment: Continuation, substitution, or combination?

Therefore, reassessment of the role of metformin involves making informed decisions regarding continuation, replacement, or combination therapy. For patients who cannot tolerate the drug or need additional glycemic control with weight reduction, newer agents such as semaglutide (a GLP-1 receptor agonist) serve as an effective alternative or additional option (Ji et al., 2021; Prasad et al., 2023). Tirzepatide, which targets both glucose-dependent insulinotropic polypeptide (GIP) and GLP-1 receptors, has demonstrated even greater effectiveness in reducing glucose levels and preserving β-cell function (Gojani et al., 2025). Cardiovascular indications further refine selection: following myocardial infarction, agents with proven cardiorenal benefits—such as SGLT2 inhibitors or GLP-1 RAs—take precedence over metformin for secondary prevention (Karwi et al., 2019). The VERIFY study further showed that early combination therapy, such as metformin with imiglitazone, provides more sustained glycemic control than metformin alone, supporting an alternative option to the stepwise escalation approach (Matthews et al., 2019; Nishiyama et al., 2023). These findings support a shift from a uniform treatment paradigm toward more flexible and evidence-based therapeutic strategies.

### 7.3 Rational combination therapy: beyond glycemic synergy

Combination regimens are increasingly designed not merely to amplify glucose-lowering potency, but to exploit mechanistic complementarity across disease pathways. The rationale is not merely based on improved glucose control; it also encompasses integrating complementary action mechanisms. Although fixed-dose combinations with sulfonylureas are extensively employed, they must be closely monitored owing to the increased risk of hypoglycemia (Bailey, 2017; Flory and Lipska, 2019). Recent studies have expanded this concept into other disease areas, including cancer. For instance, it has been reported that combining metformin with tangerine considerably inhibits tumor cell energy metabolism, thus enhancing the anticancer effects (Mdkhana et al., 2021). An alternative approach is to limit energy supply to the tumor cells by pairing metformin-mediated inhibition of glycolysis with glucose oxidase, thus creating a metabolism-based approach to cancer therapy (Meng et al., 2022). These observations indicate the evolving role of metformin in combination regimens that extend beyond glucose regulation.

### 7.4 Precision medicine integration: from population-level guidelines to individualized optimization

The future of metformin lies in predictive, biomarker-guided deployment. Optimal use will require stratification by genetic determinants (e.g., OCT1 loss-of-function variants predicting reduced hepatic uptake and diminished efficacy), microbiome signatures (e.g., baseline Akkermansia abundance correlating with glycemic response), metabolic phenotypes (e.g., insulin-resistant vs. insulin-deficient subtypes), and comorbid burden (e.g., concurrent NAFLD or heart failure). Gut microbiota modulation—via prebiotics, probiotics, or fecal microbiota transplantation—represents a promising adjuvant strategy to enhance metformin’s metabolic benefits specifically in obese, insulin-resistant individuals (Seicaru et al., 2022). Clinically, this translates to three distinct decision pathways: (i) continued metformin monotherapy for patients with excellent tolerability, preserved renal function, and isolated insulin resistance; (ii) early combination with SGLT2 inhibitors or GLP-1 RAs for those with high cardiovascular–renal risk or obesity; and (iii) substitution with newer agents for patients with intolerance, contraindications, or unmet therapeutic goals (e.g., weight loss >5%). This shift—from uniform protocols to mechanism-informed, patient-centered algorithms—will maximize therapeutic gain while minimizing adverse sequelae, ensuring metformin retains its central, albeit more precisely defined, role in metabolic medicine.

## 8 Conclusion

Metformin remains the first-line therapy for T2DM. The benefits of the drug extend beyond glycemic control, encompassing cardiovascular protection and potential antitumor effects via modulation of key metabolic pathways. Although its therapeutic efficacy in diabetes is well documented, the use of metformin is linked to adverse effects that affect multiple systems, including the nervous, digestive, reproductive, and tumor-related systems, which must be carefully considered. Consequently, controversies regarding its broader clinical application persist.

Unlike existing reviews that predominantly focus on single mechanisms or isolated effects, this work emphasizes the explicit juxtaposition of the beneficial and detrimental effects of metformin in physiological and pathophysiological contexts, with an emphasis on long-term safety in off label uses (e.g., neurodegenerative disease intervention) and in non-diabetic populations. By synthesizing conflicting evidence and highlighting context-dependent mechanisms (e.g., dose-response relationships and genetic background effects), this review fills a gap in the prior literature on integrated analysis and serves as a comprehensive reference for personalized clinical decision making.

In the future, the development of novel dual receptor agonists, such as tirzepatide and semaglutide, along with rational combination strategies involving metformin, is a promising direction for managing diabetes. These approaches can enhance therapeutic efficacy, while minimizing adverse effects via complementary and synergistic mechanisms.
